# Increasing the comprehensive economic benefits of farmland with Even-lighting Agrivoltaic Systems

**DOI:** 10.1371/journal.pone.0254482

**Published:** 2021-07-15

**Authors:** Jianan Zheng, Shoudong Meng, Xinyu Zhang, Honglong Zhao, Xiaolong Ning, Fangcai Chen, Altyeb Ali Abaker Omer, Jan Ingenhoff, Wen Liu

**Affiliations:** 1 School of Physical Sciences, University of Science and Technology of China, Hefei, Anhui, China; 2 School of Public Affairs, University of Science and Technology of China, Hefei, Anhui, China; 3 CAS Center for Excellence in Molecular Plant Sciences/Institute of Plant Physiology and Ecology, Shanghai, China; 4 Institute of Advanced Technology, University of Science and Technology of China, Hefei, Anhui, China; Bangabandhu Sheikh Mujibur Rahman Agricultural University, BANGLADESH

## Abstract

Agrivoltaic combines crop planting and electricity generation on the same land, it is considered as an opportunity to resolve the competition for land use between food and energy production. In addition to growing crops, farmers can gain electricity with the installation of agrivoltaic systems on their farmland. They can use this clean energy for agricultural production or sell it for extra income. The Chinese government considers it an important strategy for “Targeted Poverty Alleviation”. However, current methods of agrivoltaic provide uneven and low irradiance for crops, which usually results in reduced yield and low quality. In this study, an improved agrivoltaic system with a grooved glass plate has been designed, manufactured, and investigated, called Even-lighting Agrivoltaic System (EAS). Two experiments were conducted to test the effectiveness of the improvement. We measured the crops’ light environment, the crop growth process, the crop yield and quality, the electricity generation, and calculated the Land Equivalent Ratio (LER) as well as the comprehensive economic benefits on the farmland per hectare. Under the EAS, crops grew fast and the yield was similar or better than that under the natural state. By adding supplementary LED lamps into the EAS, the soluble sugar content of lettuce increased by 72.14% and the nitrate content of lettuce decreased by 21.51%. The average LER of the EAS for common vegetables was 1.64 as demonstrated in this work. Comprehensive economic benefits outperform the installation and maintenance costs, thus, the EAS can increase farmers’ income by an average of 5.14 times. The EAS provides new ideas and directions for the future development of agrivoltaic.

## 1 Introduction

Agriculture has always been an important part of human life along with the development of industrial and production levels. In the face of increasingly scarce fossil fuel reserves and climate crises such as global warming, to feed more people, it is vital to increase agricultural productivity with the help of advanced technologies [1]. Agrivoltaic was firstly proposed in 1982, which combines electricity generation and crops planting on the same farmland. There are already many other PV applications in agriculture in recent years, such as PV greenhouse, PV irrigation, PV wastewater purification, and so on [2,3]. However, most recent research on agrivoltaic systems focused exclusively on PV greenhouse, little has been done on the application of photovoltaics in field cultivation. The rudiment of the agrivoltaic system was designed by Goetzberger and Zastrow [4], the PV panels are elevated high enough above the farmland with a periodic distance between rows, which is about three times the height of the PV panels. This way crops could still grow using sunlight spread between the rows of PV panels and leveraging diffuse light. Similar designs can also be seen in PV greenhouses [5,6]. Although the current method of agrivoltaic still has some shortcomings on crop lighting, previous studies have shown that compared with conventional farming patterns, agrivoltaic creates more economic values [7,8] and performs higher productivity per land area unit [9]. As Li et al. have suggested, feed-in tariffs are less important than crop yield, thus crop planting deserves more attention in future agrivoltaic research [10].

Light is essential for plant growth, affecting plant morphology and physiology. A suitable light environment provides plants with conditions for normal morphological development and efficient photosynthesis. The current methods of agrivoltaic have a shading effect because of the PV panels above the farmland, crops cannot obtain a sufficient or uniform irradiance compared with those grown in a natural state. Homma et al. found that rice yield was reduced under the agrivoltaic systems due to the increase of shade [11]. Even when growing shade-tolerant crops such as lettuce, the experimental results also showed a yield reduction of 20 ~ 40% [9,12]. But in drylands or somewhere plants experiences severe water stress, Conventional Agrivoltaic System (CAS) helps plants reduce drought stress and maintain higher soil moisture which improves the plant biomass [13,14]. Based on the above researches, CAS increases crop yield significantly in areas where are unsuitable for plant growth such as excessive sunlight, high temperature, drought, and water shortage. However, it also leads to an evident reduction in crop yield and quality where the climate is comfortable for crop growth. This is contrary to the original intention of agriculture. And it is hard to popularize this planting method (CAS) with such agricultural deficits in common agricultural areas.

In this paper, we proposed the Even-lighting Agrivoltaic System (EAS) that considers both high yield, good quality of crops, and efficient solar power generation. As the core part of EAS, the structure of the grooved glass plate was calculated and simulated optically. An experimental base equipped with EAS was built in Fuyang city, Anhui province and a large-scale field planting experiment was performed from October 2018 to December 2019. Several common vegetables were planted for the control experiment. We recorded the crop yield and PV electricity generation and analyzed the comprehensive economic benefits of farmland per hectare. Then, we manufactured a 1:2 proportional model of EAS and designed a detailed, small-scale planting experiment in Hefei city, Anhui province. During the experiment, the light environment, plant development, crop yield, and crop quality were measured. Based on the results, we discuss the effects of EAS on crop planting and economic benefits in response to the policy of “Targeted Poverty Alleviation” in China and envision the prospect of smart agriculture represented by EAS.

## 2 Materials and methods

### 2.1 The even-lighting agrivoltaic system

**Fig 1:**
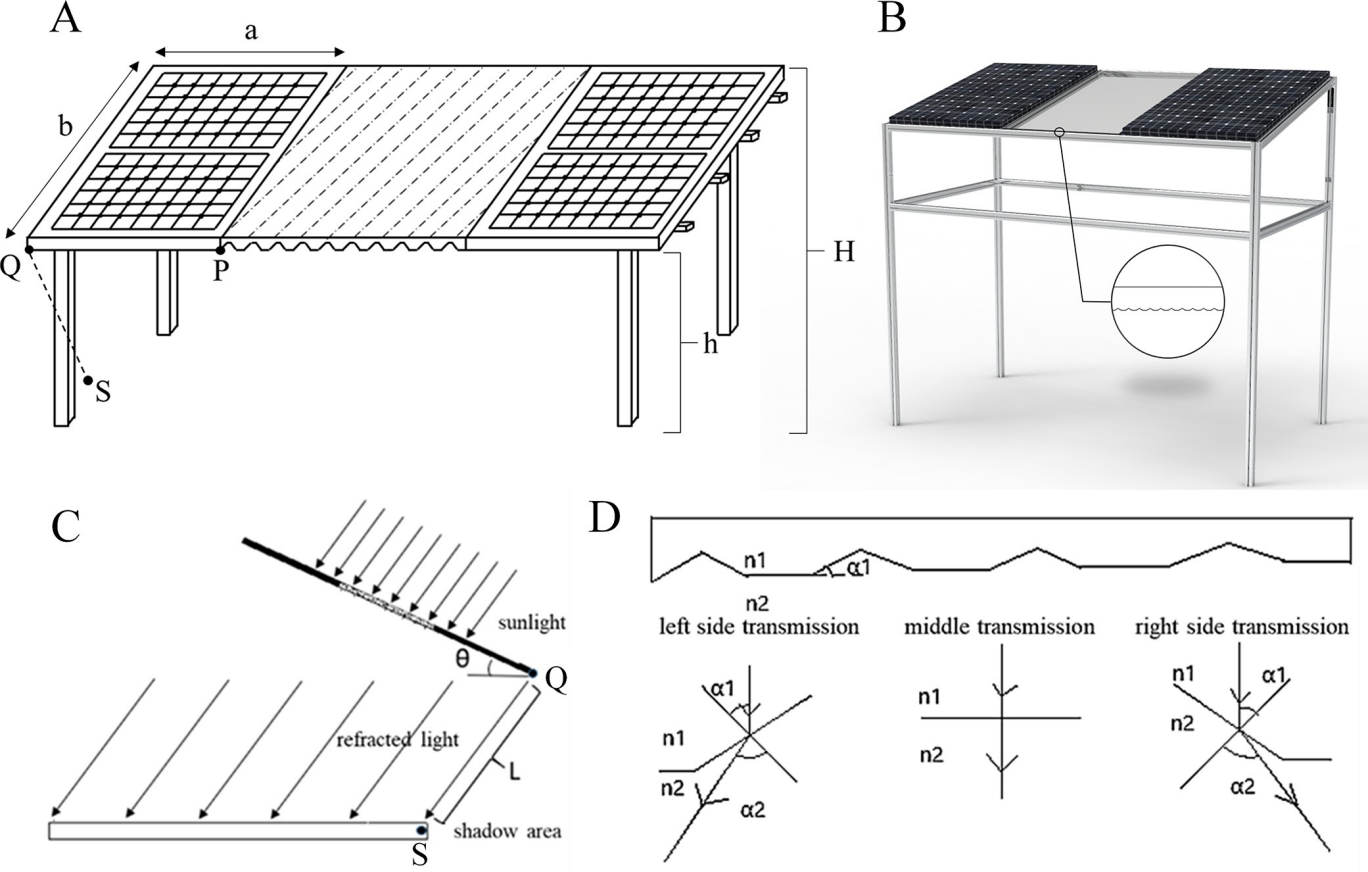
The structure of the EAS (A). The rendered output diagram of EAS structure (B). The side view of the EAS (C). The structure of the grooved glass plate (D).

The EAS was designed as shown in Fig 1A and 1B, which consists of metal brackets, regular PV panels, and a grooved glass plate. We reduced 1/3 area for PV panels deploying and designed a grooved glass plate optically to take the place. The area of the glass plate is one-third of the light-receiving area of the entire system. With the installation of the glass plate, the density of PV panel arrays is the same as conventional photovoltaic power stations and very close to the optimum design for energy production.

When the sunlight shines on the surface of the EAS, the grooved glass plate will scatter the incident light into three parts by refraction, and the shadowed areas under the system will be evenly illuminated. This way, the plants under the system gain a uniform illumination. We can adjust the transmission rate of the glass plate by changing the glass materials or the AR coatings on the surface of the glass plate to specifically alter the light intensity shining on plants.

#### 2.1.1. Geometry structure of the metal bracket

The role of the metal brackets is to elevate the PV panels and the grooved glass plates above the farmland and provide a suitable tilt angle. The support columns must be high enough to allow tall plants such as Jerusalem artichoke to grow properly under the EAS and to allow to use big agricultural machinery conveniently on the farmland. When the sunlight shines vertically on the PV panels, they will get maximum radiation and generate maximum electricity output. To collect the maximum daily solar energy, applying a sun-tracking PV system is the best solution. However, considering the expensive and complicated mechanical devices of tracking systems with often poor reliability, fixed structure installations with an optimal tilt angle are more preferable [15].

Generally, the optimal tilt angle between PV panels and horizontal plane varies with geographic latitude, utilization period (season in a year and time in a day), climate, and other atmospheric factors, i.e. dust and accumulated snow [16,17]. Empirical formulas and many accurate mathematical models have been proposed and developed. Utilizing the existing mathematical model and combining it with the latitude of Fuyang city (32°54′ N), the best tilt angle for electricity generation of a single row of PV panels is 28° ~ 30°. However, a larger tilt angle of PV panels needs larger row spacing to prevent mutual shading, which means a smaller PV installation capacity per unit area. Besides, a larger tilt angle brings stronger wind pressure, firmer metal brackets and support columns are needed, which will increase costs and reduce the return on investment. At last, considering all these issues, we determined the best tilt angle of the EAS to be 23°, which is a compromise between electricity generation and construction costs. According to the policy *Guiding Opinions of Hefei Photovoltaic Power Generation Land* issued by the local government [18], we determined the height of the EAS to be at least 2.5 m.

#### 2.1.2. Key parameters of the grooved glass plate

Refraction occurs when light travels from one medium to another medium with a different refractive index, and the relationship between the incident angle and refraction angle can be described by Snell’s law:

$$n_{1}\cdot sin\;\alpha_{1}=n_{2}\cdot sin\;\alpha_{2}$$ (1)

Based on Snell’s law and combined with the geometry structure of the metal bracket (Fig 1), the uniform illumination under the EAS can be described mathematically with the following equations:

H=h+b⋅sinθ (2)

L=h/cosθ (3)

$$\alpha =\alpha_{2}-\alpha_{1}$$ (4)

$$\alpha_{2}=sin^{-1}(n_{1}\cdot sin\;\alpha_{1})$$ (5)

$$tan^{-1}(a/L)=sin^{-1}(n_{1}\cdot sin\;\alpha_{1})-\alpha_{1}$$ (6)

$$tan^{-1}(a/L^{\prime })=sin^{-1}(n_{1}\cdot sin\;\alpha_{1}{}^{\prime })-\alpha_{1}{}^{\prime }$$ (7)

$$L^{\prime }=H/cos\;\theta$$ (8)

Where *h* is the distance between the system’s perigee and the ground, and *H* is the distance between the system’s apogee and the ground. The distance from point *Q* to point *S* on the ground (the projection point of *Q* in Fig 1C) is defined as deflection light path *L*. The deflection angle *α* represents the direction of propagation changes after the light passes through the glass plate. When the incident light reaches point *P*, the uniform illumination under the system is realized, if the light can be refracted to the left edge of the PV panel’s shadow area (point *S*). Eqs (6) and (7) describe the geometric relationship at perigee and apogee of the EAS, respectively. By solving the equations, the incident angles *α*_*1*_ and *α*_*1*_*’* are obtained. Combined with the thickness of the glass plate and the depth of the grooves, the parameters of the grooved glass plate are calculated by trigonometric relationships.

**Fig 2:**
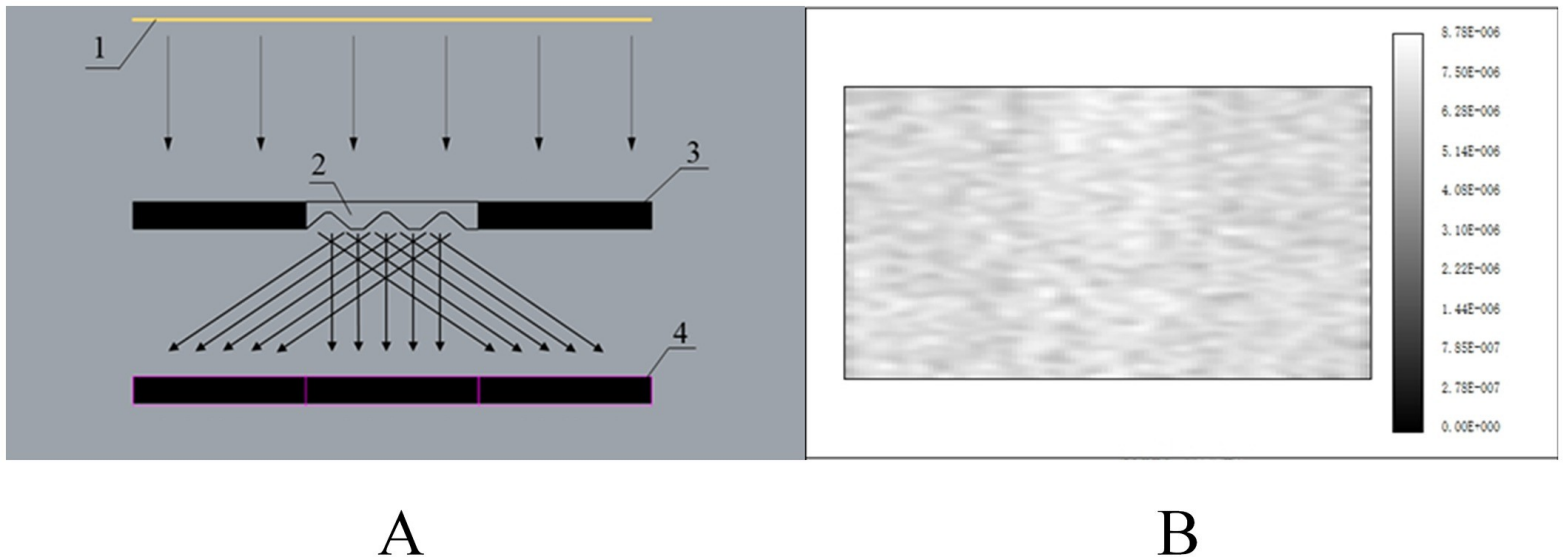
The light path of the optical simulation: 1 light source; 2 grooved glass plate; 3 PV panel; 4 illuminance detector (A). The illuminance simulation result of the glass plate (B).

To test the accuracy of the calculation for the glass plate’s parameters, we have built a model for the grooved glass plate by Solidworks 2017 (Dassault Systèmes SolidWorks Corporation, Waltham, USA) and carried out the optical simulation by importing the model file into Zemax 13 (Zemax, LLC, Washington, USA). The glass material is set to BK7, and the rectangular light source power is set to 1 W in software. The light path is shown in Fig 2A, the parallel light from the rectangular light source is scattered into three parts by the grooved glass plate, and the illuminance detector receives illuminance information in pixels.

### 2.2 Plant materials and growth conditions

This research article discusses two experiments: the first one is a detailed, small-scale experiment under semi-natural conditions performed at Institute of Advanced Technology (IAT), University of Science and Technology of China, Hefei of Anhui, China; the second one is a large-scale field planting experiment performed in Fuyang of Anhui, China.

The lettuce (*Lactuca sativa L*.) cultivar ‘(81–45) Jonction RZ’ purchased from Netherlands Rijk Zwaan Seed Seedling Group Co., Ltd. was planted at IAT. The lettuces were planted in planting pots and cultivated from March to May in 2019, totally 59 days. We prepared 32 planting pots, and each one contained 9 plants. During the experiment, plants in each treatment were kept at the same conditions of watering and fertilization.

For the large-scale field planting experiment in Fuyang city, experimental plants including broccoli (*Brassica oleracea L*.*var*.*italic Planch*.), shallot (*Allium*. *fistulosum L*.*var*. *gigantum Makino*), garlic sprouts (*Allium sativum L*.), garlic (*Allium sativum L*.), rape (*Brassica napus L*.), broad bean (*Vicia faba L*.), and Jerusalem artichoke (*Helianthus tuberosus L*.) were leveraged. The cultivation information is shown in Table 1. During the growth period, plants were fertilized and watered accordingly to their growth states. Plants in different treatments stayed at the same condition of watering and fertilization.

**Table 1:** The cultivation information and average prices of crops in the large-scale field planting experiment.

Crop	Planting time	Harvest time	Planting density	Planting area (m^2^)	Average prices in Anhui Province 2019 (CNY/kg)
Broccoli	October 2018	December 2018	4 plants/m^2^	100	3
Shallot	October 2018	March 2019	25 clusters/m^2^	150	2
Garlic sprouts	October 2018	December 2018	50 plants/m^2^	250	6
Garlic	October 2018	March 2019	50 plants/m^2^	250	5
Rape	October 2018	March 2019	4 plants/m^2^	100	5
Broad bean	October 2018	March 2019	10 plants/m^2^	250	4
Jerusalem artichoke	April 2019	November 2019	4 holes/m^2^	150	2

The growth time of the crops in this table is about 2.5 months, 5.5 months, 3 months, 5.5 months, 5.5 months, 5.5 months, 7 months respectively.

### 2.3 Design of experiments

**Fig 3:**
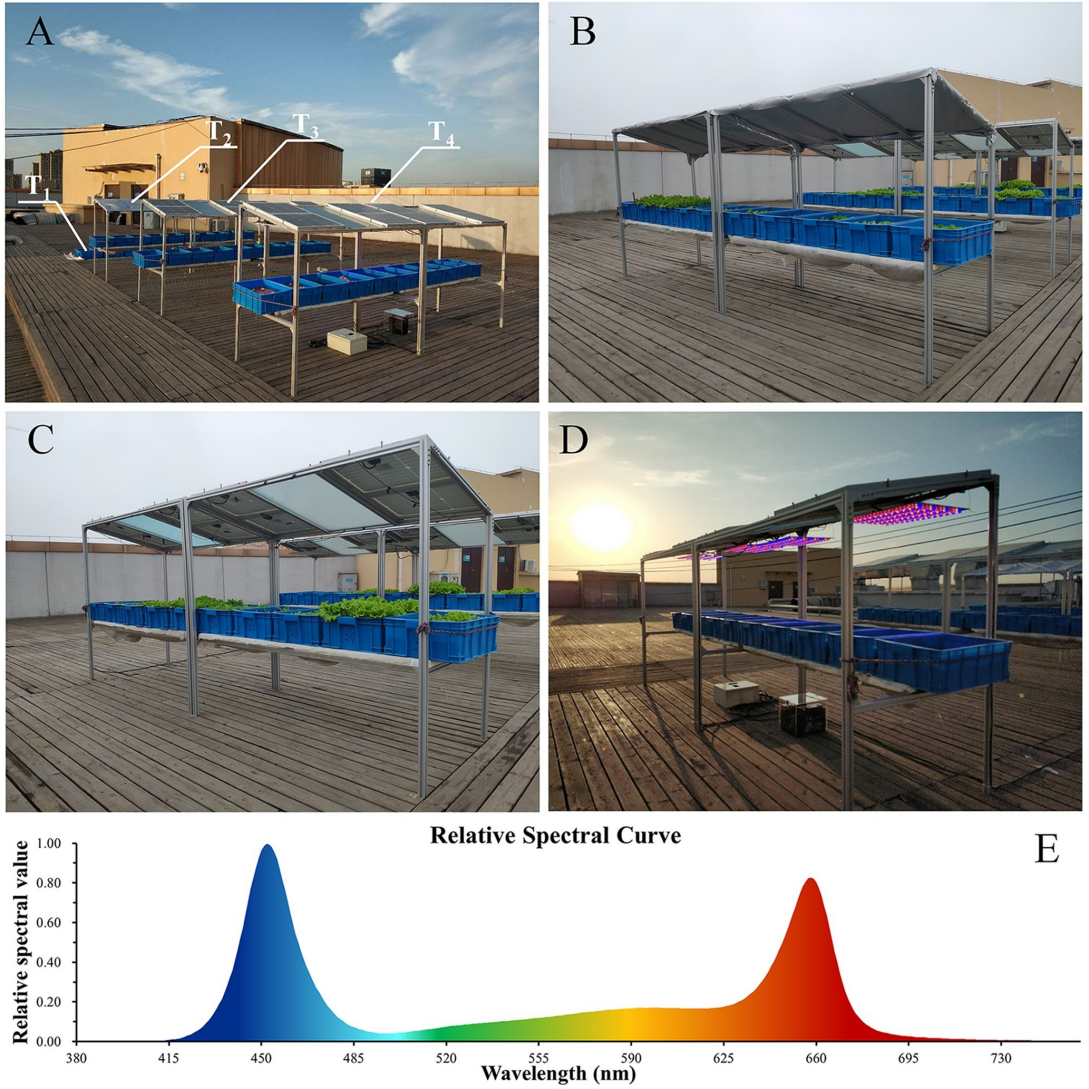
Photographs of the small-scale experimental facilities of the four treatments (T_1_, T_2_, T_3_, T_4_ at IAT, USTC, Hefei of Anhui, China). Overall photograph of the four treatments (A). Photograph of T_2_ (The shading cloth was used to simulate the shading effect of PV panels whose shading effect is the same as the PV panels after testing.) (B). Photograph of T_3_ (C). Photograph of T_4_ (The supplementary LED lamps were turned on to show and test the system function.) (D). The light spectrum of the supplementary LED lamps (E).

Four treatments were set up in the detailed, small-scale experiment (Fig 3). T_1_: The plants grew in a natural state. T_2_: The plants grew under the CAS. T_3_: The plants grew under the EAS. T_4_: The plants grew under the EAS equipped with supplementary LED lamps, which provided plants an extra 2 h light time every day compared to other treatments. The LED lamps were powered by part of the electricity converted by the PV panels in T_4_. They provided a one-hour illumination before sunrise and after sunset, respectively. The LED lamps consisted of a combination of red LED chips (660 nm), blue LED chips (450 nm), and white LED chips (color temperature 3000 K), and the light spectrum was designed by an artificial climate chamber which was suitable for lettuce growth (Fig 3E) [19].

**Fig 4:**
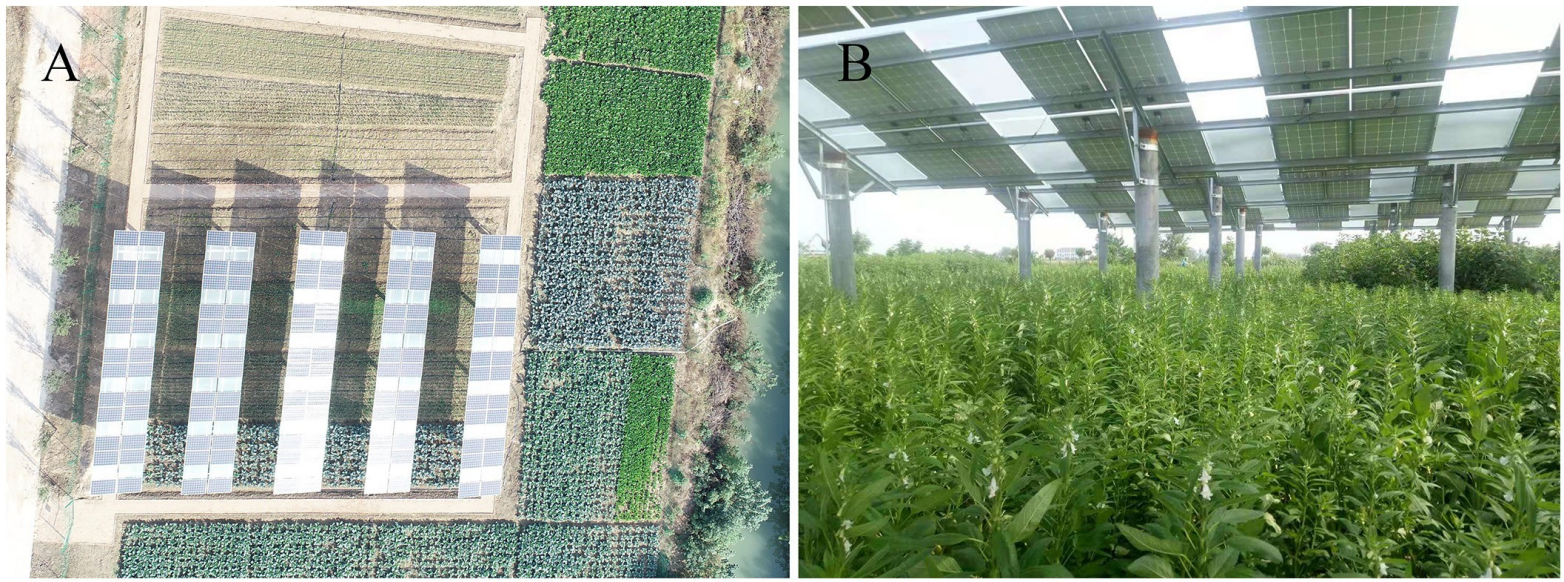
Photographs of the large-scale experimental facilities of the two treatments (T_a_, T_b_ in Fuyang of Anhui, China). Aerial photograph of Fuyang Experimental Base (A). Details photograph under the EAS (B).

Two treatments were set up in the large-scale field planting experiment (Fig 4). T_a_: Plants grew on farmland in a natural state; T_b_: Plants grew under the EAS. The experimental base covered an area of 480 m^2^.

### 2.4 Light environment and electricity generation measurement

Photosynthetic photon flux density (PPFD) represents the number of photons absorbed by plants in the photosynthetically active band (400 nm ~ 700 nm), and it was measured by an illuminometer (LI-250A Light Meter and LI-190R Quantum Sensor, LI-COR, Lincoln, Nebraska, USA). The light environments beside the lettuce leaves (under the CAS and EAS), on PV panels, and the ground were measured respectively. From 7:00 to 19:00, the PPFD was recorded every 30 minutes, each position was measured for 15 s to obtain an average value.

The total installed capacity of the EAS in Fuyang experimental base was 35 kW. An energy meter was used to measure the electricity generation from October 2018 to December 2019. The average daily power generation per month was recorded and the power generation per hectare was calculated.

### 2.5 Crop growth, yield, and quality measurement

#### 2.5.1. Crop growth measurement

To explore the effect of different light conditions on plant growth and development in each treatment, four weeks after lettuce’s sowing, the maximal width of each plant was measured and the leaf number of each plant was counted once a week. Cotyledons and withered leaves were not included in the leaf number. 36 plants (in four planting pots) were randomly selected and measured in each treatment.

#### 2.5.2. Crop yield measurement

58 days after lettuce’s sowing, 20 plants were randomly chosen from each treatment. After removing the roots and the yellow senescent leaves, the fresh weight of each plant was measured. Afterward, the plants were put into an electric blast drying oven (DGT-G82A, Hefei Darth Carter Biotechnology Co., Ltd., Hefei, Anhui, China) and dried at 105°C for 30 minutes, then the temperature of the oven was set to 85°C until the plants reach the constant weight. Both the fresh weight and dry weight were measured using an electronic balance (OHAUS Scout SE, OHAUS, New Jersey, USA). And the water content of each plant was calculated by the following equation:

$$water\;content=\frac{fresh\;weight-dry\;weight}{fresh\;weight}\times 100\%$$ (9)

To compare the crop yield in the farmland between T_a_ (plants grown in a natural state) and T_b_ (plants grown under the EAS), plants were sampled randomly from each treatment and measured for their fresh weight or dry weight.

#### 2.5.3. Crop quality measurement

Lettuce is a popular and largely consumed vegetable with rich nutrition. It is important to estimate the nutrition level of lettuce under different treatments. Soluble protein was determined with the Coomassie brilliant blue G-250 staining method [20]; soluble sugar content was determined with the copper reduction iodometry method [21]; vitamin C content was determined with the high-performance liquid chromatography method [22]; nitrate content was determined with the ion chromatography method [23].

### 2.6 The comprehensive economic benefits of EAS

The comprehensive economic benefits of EAS consist of the installation and maintenance costs, the electricity generation benefits and the crops harvest benefits. To accurately evaluate the comprehensive benefits of planting different crops, we considered the time required to plant different crops and the amount of electricity generated by the PV panels during the crop growth. We assume that the service life of the EAS is *N* years, the installation cost of EAS is *I*_*C*_ CNY/W, the maintenance cost is *M*_*C*_ CNY/W, the installed capacity of PV per hectare is *P* W. For crop X, we assume that the crop yield is *Y*_*X*_ kg/ha, the price of crop X is *P*_*X*_ CNY/kg, the electricity generation is *E*_*X*_ kW∙h during the growth time *T*_*X*_, the price of electricity is *P*_*E*_ CNY/kW∙h. The comprehensive economic benefits of EAS is defined as:

$$\text{Comprehensive}\;Economic\;Benefits\;of\;Crop\;X=\;Y_{X}\cdot P_{X}+E_{X}\cdot P_{E}-\frac{I_{C}+M_{C}\cdot N}{N}\cdot P\cdot T_{X}$$ (10)

### 2.7 The performance of land use

Land Equivalent Ratio (LER) is an indicator to access the land productivity of mixed cultivated patterns, which is suitable to evaluate the performance of EAS [7]. It is defined as:

$$LER=\frac{Crop\;Yield_{EAS}}{Crop\;Yield_{natural\;stste}}+\frac{Electicity_{EAS}}{Electricity_{PV\;Station}}$$ (11)

If LER > 1, the EAS is more effective than the pattern of separately planting crops and building solar power stations on the same land area.

### 2.8 Statistics analysis

All measurements were replicated three times or more to ensure the results reliable. The data were analyzed using one-way ANOVA by SPSS 25 (IBM, New York, USA), and an LSD’s multiple range test was used to determine differences between treatments for all investigated traits. P-values smaller than 0.05 were considered as significant differences for pairwise comparisons.

## 3 Results

The grooved glass plate in EAS improved the irradiation collected by crops in one day by 47.38% compared with those in CAS and provided a better light environment for crop growth. Crops’ growth rate in EAS is similar to the natural state. The yield of experimental crops except broccoli and rape fell by approximately 5%, and the yield of Jerusalem artichoke increased by 23%. Combined with the PV electricity generation, the EAS increased farmers’ average income by 5.14 times and performed a high LER (averagely 1.64). Interestingly, we also found that supplementing light with a specific spectrum for a short period can significantly change the quality of crops.

### 3.1 Optical simulation of the grooved glass plate

Through the software’s ray tracing algorithm, we obtained the illuminance simulation results (Fig 2B). The optical power, which the detector received was 0.322 W, and the optical power received by the three shadow parts under the glass plate was 0.104 W, 0.114 W, and 0.103 W from left to right respectively. The grooved glass plate achieved a uniform light-scattering with little difference.

### 3.2 Light environment under different conditions

**Fig 5:**
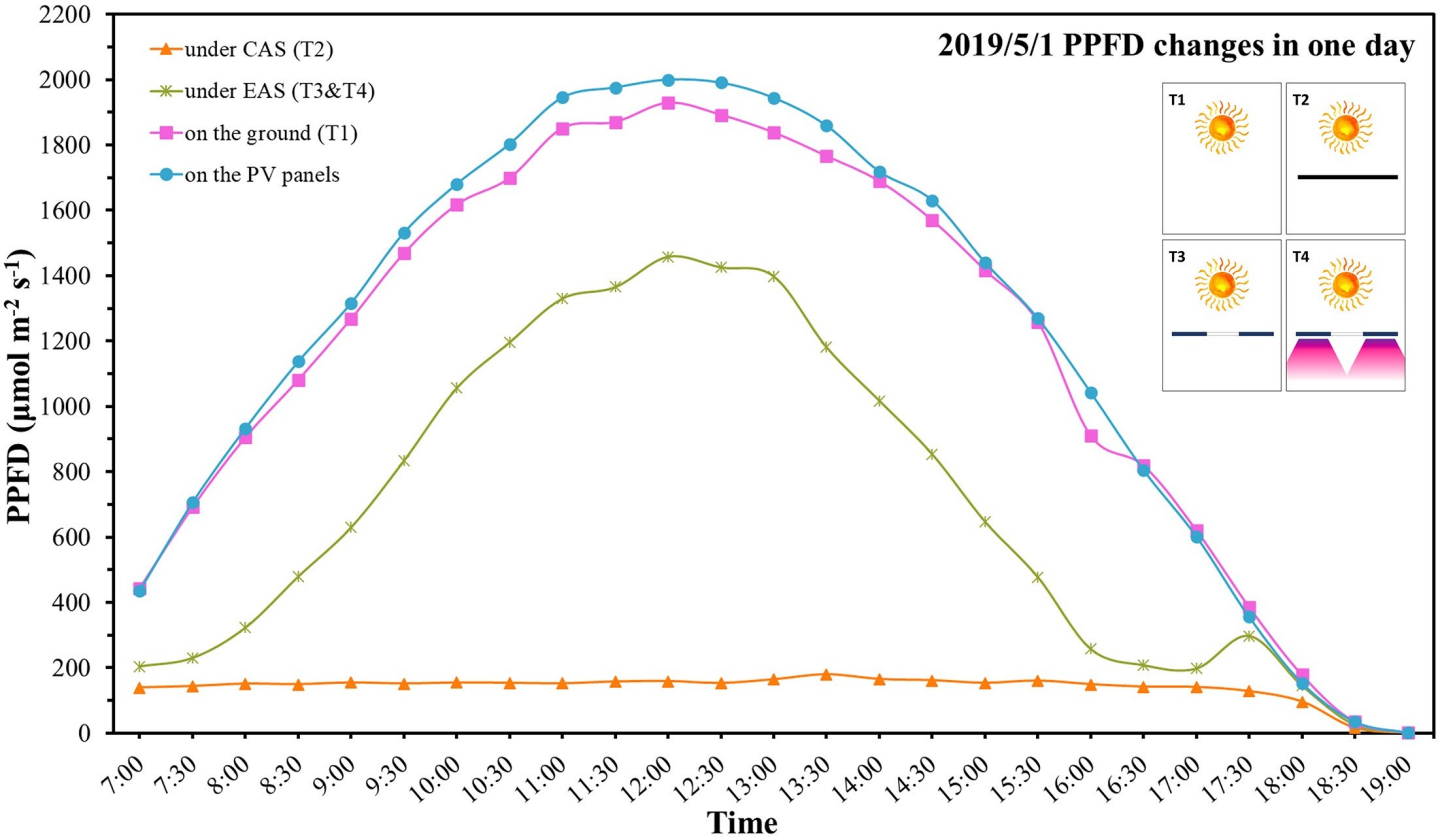
Variation of the typical light environment (PPFD) of different treatments over time. The detailed caption of legends is shown in Section 2.4.

Light environments in different treatments are shown in Fig 5. The variation tendency of PPFD under the EAS (T_3_ & T_4_: green curve) was the same as that on the ground (T_1_: purple curve) and the PV panels (blue curve). However, the PPFD under the CAS (T_2_: orange curve) was very low during the daytime. Due to the shading effect of the PV panels, only diffuse light in the air reached the leaf surface, so the average PPFD in T_2_ was 152.76 μmol m^-2^ s^-1^. The solar irradiance was strongest at noon, and the PPFD curve reached its peak. The maximum PPFD was 2000 μmol m^-2^ s^-1^, 1928.5 μmol m^-2^ s^-1^, and 1458 μmol m^-2^ s^-1^ on the PV panels, in T_1_, and T_3_ & T_4_, respectively. Because of the suitable tilt angle, the irradiation collected during the day on the surface of the PV panels was 3.87% more than that on the ground. The irradiation collected in T_3_ & T_4_ was 40.87% less than T_1_, and the irradiation collected in T_2_ was 88.25% less than T_1_. Compared with the CAS, the EAS increased the irradiation that crops received in one day by 47.38%.

At sunset, the angle between sunlight and the horizontal plane is small, sunlight is directly incident under the EAS, so there is a small “bump” on the right side of the green curve in Fig 5. We also measured the light environment under the supplementary LED lamps in T_4_. The PPFD provided by LED lamps was approximately 700 μmol m^-2^ s^-1^.

### 3.3 Crop measurement

#### 3.3.1. Crop growth under different conditions

**Fig 6:**
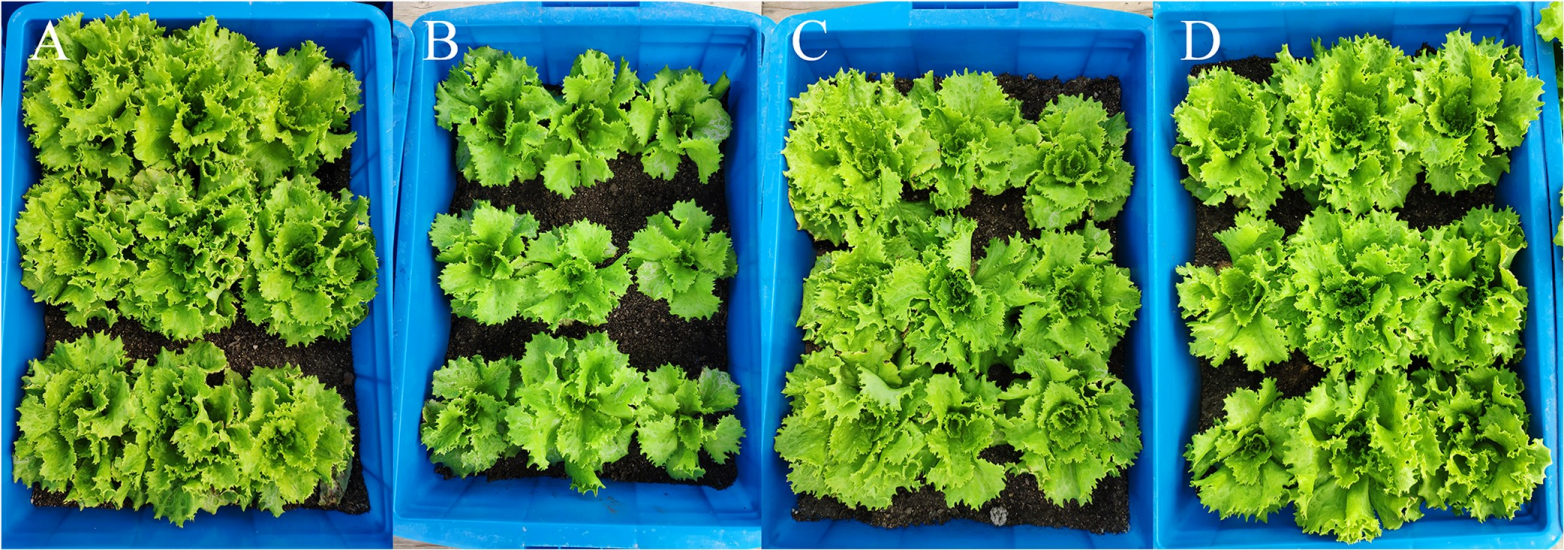
Photographs of the morphological differences of the four treatments on the 58^th^ day after sowing. T_1_ (A), T_2_ (B), T_3_ (C), T_4_ (D).

In Tables 2 and 3, the number of leaves and the plant’s maximal width for different light conditions was the same at the beginning of the experiment (30 days). But on the 58^th^ day after sowing, the number of leaves trend was T_1_ > T_3_ = T_4_ > T_2_; the plant’s maximal width trend was T_3_ > T_1_ = T_4_ > T_2_. The samples in T_1_, T_3_, T_4_ have 32.34%, 21.51%, 18.78% more leaves than those in T_2_, respectively. For the maximal width of lettuces, samples in T_1_, T_3_, T_4_ are 7.38%, 12.67%, 5.71% larger than those in T_2_, respectively. The low light environment causes lettuces in T_2_ to grow slowly. They need additional 7 days or more to grow to the similar level as other treatments, which increases the land occupation time. In addition, the morphology of plants in each treatment had different characteristics. For T_1_ treatment, which received the strongest light, the samples were shorter but had more leaves; the leaves were thick and strong, the leaf color was dark green. For T_2_ treatment, which received the weakest light, the samples were taller but had fewer leaves; the leaves were long and thin, usually weak, and the leaf color was emerald. Plants grown in T_3_ and T_4_ were between the two extremes (Fig 6).

**Table 2:** Effect of different light conditions on the number of lettuce leaves.

Age of plants (Days)	30	37	44	51	58
Treatment					
T_1_	4.81 ± 0.23a	7.86 ± 0.61a	10.22 ± 0.52a	13.39 ± 0.89a	16.00 ± 1.77a
T_2_	4.53 ± 0.11a	5.95 ± 0.34c	8.06 ± 0.64b	10.53 ± 0.33c	12.09 ± 0.83c
T_3_	4.89 ± 0.52a	6.80 ± 0.31b	9.91 ± 0.81a	12.70 ± 0.82ab	14.69 ± 0.52ab
T_4_	4.89 ± 0.60a	6.74 ± 0.64b	9.33 ± 1.13a	12.03 ± 1.04b	14.36 ± 0.88ab

The data shown are the mean values of 4 replicates of planting pots per treatment (each planting pot contained 9 plants). Different letters indicate significant differences at P < 0.05, using LSD’s Multiple Range Test.

**Table 3:** Effect of different light conditions on the maximal width of lettuces (mm).

Age of plants (Days)	30	37	44	51	58
Treatment					
T_1_	49.75 ± 1.19a	76.92 ± 6.45a	102.81 ± 7.15ab	116.53 ± 10.76ab	130.89 ± 9.56ab
T_2_	48.03 ± 1.62a	70.70 ± 2.67a	93.08 ± 7.33b	108.92 ± 4.33b	121.89 ± 5.73b
T_3_	50.17 ± 3.72a	81.52 ± 8.50a	106.77 ± 8.45a	123.82 ± 5.84a	137.33 ± 10.78a
T_4_	50.25 ± 1.96a	69.51 ± 11.51a	93.97 ± 10.64ab	111.22 ± 11.83ab	128.85 ± 9.82ab

The data shown are the mean values of 4 replicates of planting pots per treatment (each planting pot contains 9 plants). Different letters indicate significant differences at P < 0.05, using LSD’s Multiple Range Test.

#### 3.3.2. Crop yield under different conditions

As shown in Table 4, the average fresh weight of lettuce rank under different light conditions was T_3_ = T_4_ = T_1_ > T_2_; the average dry weight of lettuce rank was T_1_ = T_3_ = T_4_ > T_2_. Under the CAS (T_2_), the value of the average fresh weight and dry weight was reduced by 53.5% and 60.5% compared to those in T_1_. It indicated that the conventional agrivoltaic method resulted in more than 50% lettuce yield reduction which is a disaster for agricultural production. There were no significant yield differences among T_1_, T_3,_ and T_4_. It suggests that plants under the EAS gain a similar yield compared with those in a natural state. It’s worth mentioning that samples grown in T_1_ had significantly lower water content, which made lettuces taste bad.

**Table 4:** Different light conditions and their effect on lettuce’s physiological parameters.

Treatments	T_1_	T_2_	T_3_	T_4_
Parameters	Natural State	Conventional Agrivoltaic System	Even-lighting Agrivoltaic System	Even-lighting Agrivoltaic System with LED lamps
Irradiation collected during daytime (%)	100	11.75	59.13	59.13
Average leaf number before harvest	16.00 ± 1.77a	12.09 ± 0.83c	14.69 ± 0.52ab	14.36 ± 0.88ab
Average maximal width of lettuce before harvest (mm)	130.89 ± 9.56ab	121.89 ± 5.73b	137.33 ± 10.78a	128.85 ± 9.82ab
Average fresh weight (g)	17.89 ± 10.01a	8.32 ± 4.57b	18.44 ± 5.89a	18.32 ± 9.47a
Average dry weight (g)	1.57 ± 0.78a	0.62 ± 0.33b	1.38 ± 0.43a	1.35 ± 0.63a
Average water content (%)	90.99 ± 0.008b	92.52 ± 0.007a	92.44 ± 0.006a	92.46 ± 0.009a
Soluble protein (mg/g FW)	0.178 ± 0.030a	0.219 ± 0.027a	0.177 ± 0.019a	0.176 ± 0.017a
Soluble sugar (g/100 g FW)	0.884 ± 0.020c	1.082 ± 0.045a	0.585 ± 0.027d	1.007 ± 0.050b
Vitamin C (mg/100 g FW)	8.015 ± 0.244c	10.002 ± 0.121a	9.081 ± 0.094b	4.708 ± 0.188d
Nitrate (mg/kg FW)	3317.830 ± 56.010b	4511.006 ± 165.433a	4432.343 ± 123.938a	3478.943 ± 105.133b

The average fresh weight, dry weight, and water content are the mean values of 20 replicates per treatment. The soluble protein is mean values ± SD of 3 replicates per treatment. The soluble sugar, vitamin C, and nitrate are mean values ± SD of 4 replicates per treatment. Different letters indicate significant differences at P < 0.05, using LSD’s Multiple Range Test.

In the large-scale field planting experiment in Fuyang city (Fig 4), crop yields harvested throughout 2019 are shown in Table 5. Overall, the single yield of plants in T_b_ was a little lower than that in T_a_. The yields of broccoli and rape declined by 9% and 11% respectively. Yields for shallot, garlic sprouts, garlic, and broad bean decreased by 2%, 6%, 4%, and 6%. However, the yield of Jerusalem artichoke grown under the EAS increased significantly by 23%. It shows that the EAS has great advantages for the cultivation of shade-tolerant crops represented by Jerusalem artichoke. And the yield reduction of other shade-intolerant crops is within the acceptable range.

**Table 5:** Yield per single plant and yield per hectare of two cultivation methods.

Crop	Yield in T_a_ (kg)	Yield in T_b_ (kg)	Yield per hectare in T_a_ (kg/ha)	Yield per hectare in T_b_ (kg/ha)
Broccoli	0.7400	0.6700	25530.00	23115.00
Shallot	0.2048	0.2000	51225.60	50025.00
Garlic sprouts	0.0384	0.0360	19209.60	18009.00
Garlic	0.0386	0.0372	15640.50	15055.80
Rape	0.0204	0.0182	2037.90	1818.15
Broad bean	0.0571	0.0540	5706.90	5391.75
Jerusalem artichoke	0.8525	1.0500	29411.25	36225.00

The yield of broccoli, shallot, garlic sprouts, Jerusalem artichoke is represented by the average fresh weight of a single plant; the yield of garlic and broad bean is the average dry weight of a single plant; the yield of rape is the average fresh weight of thousand-seed. T_a_: In the natural state; T_b_: Under the EAS.

#### 3.3.3. Crop quality under different conditions

As shown in Table 4, there are no significant differences among the four treatments for soluble protein content. The soluble sugar and vitamin C content in T_2_ were significantly higher than those in T_1_, T_3_, and T_4_. The nitrate content in T_2_ is similar to that in T_3_, which is higher than that in T_1_ and T_4_. Compared with T_1_, vitamin C and nitrate content in T_3_ increased by 13.30% and 33.59%, respectively, and the soluble sugar content of T_3_ was 33.82% lower than that in T_1_. Although there was a small difference in the light environment between T_3_ and T_4_, the values of soluble sugar, vitamin C, and nitrate content were all significantly dissimilar. In T_4_ treatment, the soluble sugar content increased by 72.14%, and the nitrate content decreased by 21.51%. However, the vitamin C content in T_4_ was lower than that in T_3_ by 48.16%. In summary, the rank for soluble sugar content in lettuce was T_2_ > T_4_ > T_1_ > T_3_; for vitamin C, it was T_2_ > T_3_ > T_1_ > T_4_; for nitrate content, it was T_1_ = T_4_ < T_3_ = T_2_.

### 3.4 Electricity generation and comprehensive economic benefits

The electricity generation data from October 2018 to December 2019 are shown in Table 6. We estimated the installation and maintenance costs for the EAS in Table 7. It is reported that the service life of a PV system can reach 25 years or even longer [24]. In this study, we choose 15 years as a conservative estimate. Based on *Village-level Poverty Alleviation Photovoltaic Power Station Subsidy Standards* issued by the local government, the selling price of electricity is 0.85 CNY/kW·h [25]. Combining the data of crop yields in T_b_ (Table 5), the prices of crops, and the growth periods (Table 1), we calculated the comprehensive economic benefits per hectare according to Eq (10). Farmers’ income is increased averagely by 5.14 times with the installation of EAS (Table 8).

**Table 6:** Monthly electricity generation of the EAS.

Date	Monthly Electricity Generation (kW·h)	Monthly Electricity Generation per hectare (kW·h)	Days	Daily Average Electricity Generation (kW·h)	Daily Average Electricity Generation per hectare (kW·h)
2018–10	4130	86042.10	31	133.23	2775.55
2018–11	2319	48307.53	30	77.29	1610.25
2018–12	1689	35182.47	31	54.48	1134.92
2019–1	1916	39922.07	31	61.81	1287.81
2019–2	735	15312.58	28	26.25	546.88
2019–3	4340	90417.12	31	140.00	2916.68
2019–4	3780	78750.39	30	126.00	2625.01
2019–5	4506	93880.68	31	145.36	3028.41
2019–6	4244	88411.90	30	141.46	2947.06
2019–7	4743	98802.58	31	152.98	3187.18
2019–8	4446	92622.86	31	143.42	2987.83
2019–9	4033	84018.65	30	134.43	2800.62
2019–10	3106	64713.87	31	100.20	2087.54
2019–11	2651	55234.65	30	88.38	1841.16
2019–12	2564	53411.73	31	82.70	1722.96

**Table 7:** The installation and maintenance costs of EAS.

Type of cost		Price
Materials	Grooved glass plate	0.32 CNY/W
	Photovoltaic module	1.4 CNY/W
	Cable	0.2 CNY/W
	Bracket	0.6 CNY/W
	Inverter	0.3 CNY/W
Construction		0.6 CNY/W
Foundation		0.4 CNY/W
Operation and Maintenance		0.05 CNY/W∙Year

**Table 8:** Economic benefits per hectare of land in two cultivation methods.

Treat-ment	Crop	Economic value of crops per hectare (CNY)	Economic value of electricity generation per hectare (CNY)	Amortized cost during planting time (CNY)	Total income per hectare (CNY)
T_a_	Broccoli	76590.00	0	0	76590.00
	Shallot	102451.20	0	0	102451.20
	Garlic sprouts	115257.60	0	0	115257.60
	Garlic	78202.50	0	0	78202.50
	Rape	10189.50	0	0	10189.50
	Broad bean	22827.60	0	0	22827.60
	Jerusalem artichoke	58822.50	0	0	58822.50
T_b_	Broccoli	69345.00	129149.73	46281.83	152212.90
	Shallot	100050.00	229479.01	101820.03	227708.98
	Garlic sprouts	108054.00	144102.28	55538.20	196618.08
	Garlic	75279.00	229479.01	101820.03	202937.98
	Rape	9090.75	229479.01	101820.03	136749.73
	Broad bean	21567.00	229479.01	101820.03	149225.98
	Jerusalem artichoke	72450.00	501026.59	129589.13	443887.46

### 3.5 Land equivalent ratio

**Fig 7:**
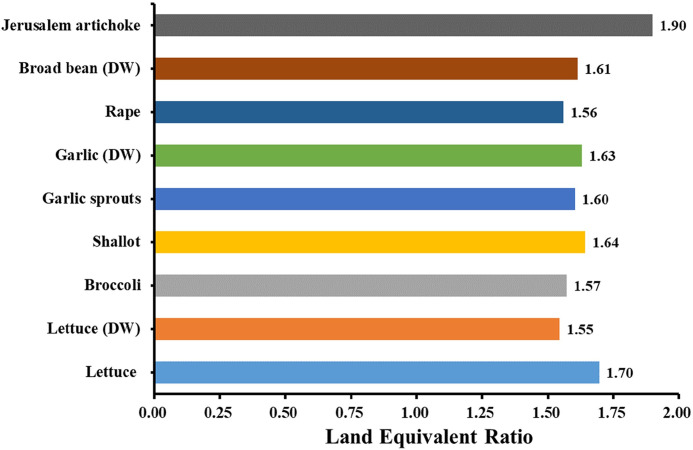
Land equivalent ratio of EAS for experimental plant varieties. DW: Dry weight.

Land Equivalent Ratios for experimental plant varieties were computed according to Eq (11), which gives the relative land area required to produce the same biomass and electricity. Because of the structure of EAS, the $\frac{Electicity_{EAS}}{Electricity_{PV\;Station}}$ equals to $\frac{2}{3}$. Thus, the more crop yield we harvested under the EAS, the higher LER we obtained. Excitingly, LER values always exceeded 1, averagely 1.64, regardless of the plant variety (Fig 7). It means the EAS has more efficiency in land use than under separate production patterns.

## 4 Discussion

Treatments in the detailed, small-scale experiment: T_1_: The plants grew in a natural state; T_2_: The plants grew under the Conventional Agrivoltaic System (CAS); T_3_: The plants grew under the Even-lighting Agrivoltaic System (EAS); T_4_: The plants grew under the EAS equipped with supplementary LED lamps.

Treatments in the large-scale field planting experiment: T_a_: Plants grew on farmland in a natural state; T_b_: Plants grew under the EAS.

Detailed experimental settings are described in Section 2.3.

### 4.1 Effects of EAS on plants’ growth, yield, and quality

Light plays an important role in the life of plants: it is the energy source for carbon dioxide fixation, and it is a vital signal to regulate various physiological processes and drives many light-dependent biochemical reactions [26]. The growth, yield, and quality of plants can be considerably different under different light conditions.

By adding grooved glass plates, EAS increased the irradiation collected by lettuce by 47.38% compared with the CAS during daytime (Fig 5). Due to the changes of daily sun azimuth, crops in CAS receive severely fluctuating light conditions where shading alternated at varying rates, and some of the crops must grow in the shade of PV panels all the time. The fluctuating light condition or low light intensity results in a longer growth period and poorer crop yield, which is reflected in fewer leaf numbers and less plant’s maximal width (Tables 2 and 3). The plant’s maximal width basically reflected the leaf length. To capture more photosynthetic photons, lettuces in T_3_ have longer leaves than those in T_1_. However, the plant’s maximal width in T_4_ was suppressed at the same level as in T_1_. It means that properly extending the light time has the same effect as increasing the light intensity [27]. By providing a suitable light environment, lettuces under EAS (T_3_ & T_4_) maintain a normal natural growth rate and there are no significant yield differences among T_1_, T_3,_ and T_4_. Combined with the results of large-scale field planting experiment, shade-tolerant plants represented by Jerusalem artichoke and lettuce obtained similar or more yield than those in a natural state; the yield of shade-intolerant plants that do not require too much light represented by shallot, garlic sprouts, garlic, and broad bean decreased by approximately 5%; the yield of shade-intolerant plants that require much light represented by broccoli and rape decreased by approximately 10%. Common crops planted under EAS obtained an acceptable yield, which proves that EAS has the potential to realize unaffected crop growth and electricity generation simultaneously.

In addition to crop yield, the crop quality in different treatments showed significant differences (Table 4). The values of these four crop quality parameters in T_2_ were significantly higher. A possible explanation for this is that the unsuitable environment stressed the plant growth and stimulated the synthesis of the plant’s secondary metabolites [28]. Bian et al. reported that soluble sugar and vitamin C content increased, and nitrate content substantially decreased as the light intensity increased in a certain range [29]. Zhou et al. also reported that extending the light time had the same effect as increasing light intensity on hydroponic lettuce [30]. Nitrate is a harmful substance with a carcinogenic action [31]. Because of the extra 2 h light time provided by supplementary LED lamps, the nitrate content in T_4_ dropped to the same level as in T_1_, and it was 21.51% lower than that in T_3_. From the data, we found that soluble sugar content in T_4_ was higher than that in T_1_ and T_3_, which indicates that the spectral composition of red, blue, and white light promotes soluble sugar synthesis. This is confirmed and supported by the results from Liandong et al. and Lin et al. [32,33]. The vitamin C content in T_4_ is lower than that in T_3_, which is contrary to previous research results that extending the light time can increase the vitamin C content. We considered that the specific spectrum and longer light time used in T_4_ inhibited vitamin C biosynthesis. Liu and Yang found that the vitamin C synthesis of bean seedlings was severely inhibited with a supplementary UV-C light for one hour at night [34], which might support our hypothesis. The spectral composition of artificial light has a great influence on plants’ nutritional quality [35], and we need to optimize the spectrum further to avoid decreasing vitamin C content.

### 4.2 High land equivalent ratio and microclimate changes in EAS

With the rapid population growth, the land-use competition between food and energy production is dramatically increasing. The EAS will be a new solution with its high LER. The average LER of all the experimental plant varieties in our study was 1.64 (Fig 7), which indicates that to obtain the same biomass and PV electricity as EAS in a separate production method, approximately 1.64 times land area is required. For some shade-tolerant crops represented by Jerusalem artichoke and lettuce, the LER value reached an astonishing 1.9 and 1.7. High LER lays the foundation for high economic benefits.

Although the grooved glass plates improve the light intensity under the PV panels, the light intensity is still weaker compared to direct sunlight. As we hypothesize, the leaf temperature under the EAS will be lower than under the natural state, hence the transpiration rate and soil water evaporation rate will be also low. The agrivoltaic system has shown another advantage of protecting crops from light damage and reducing water evaporation [13,14]. Marrou et al. reported that shading irrigated vegetable crops with PVPs (photovoltaic panels) allowed saving 14% ~ 29% of evapotranspiration water [36], which depended on the level of shade created and the crop grown in the system. For our research, the microclimate changes under EAS need further systematic investigation.

### 4.3 Significance of EAS for “Targeted Poverty Alleviation”

With uneven regional development and the large rural populations in poverty, the Chinese government has issued many documents and policies to support the development of rural areas [37–39]. According to the statistics, agricultural greenhouse gas emissions have been estimated at 11% of China’s national emissions [40]. *Photovoltaic Poverty Alleviation* was proposed for these problems, which is consistent with the concept of agrivoltaic, helps increase farmer’s income, and realizes clean power supply and environmental protection. Nacer et al. reported that a PV system with an installed capacity of 23 kW can mitigate greenhouse gases by 554 t over the system lifetime [41], a case study site in Central West NSW also proved agrivoltaic system can deliver significant social and environmental benefits on a local level [42].

In our study, the EAS showed great progress in agricultural production (Table 8). The economic benefits increased through solar power generation vary from 75,623 to 385,065 CNY depending on the crop variety planted per hectare. Farmers’ income is increased averagely by 5.14 times. Based on low installation costs and long service life, the EAS has a short return on investment period. It also does not reduce arable land areas, thus the EAS can ease the food crisis. With the installation of EAS on their farmland, farmers can carry out normal agricultural activities, obtain clean electricity simultaneously on the same land. The ease of integration, high compatibility, and high economic benefits remove the barriers on the way to increasing the adoption of EAS among farmers [43]. In the future, EAS can provide a power supply for other necessary farmland facilities, such as supplementary lighting lamps, drip irrigation, and agricultural weather stations, which lays the foundation for future smart agriculture. The promotion of EAS in poverty-stricken areas through government investments and cooperation of photovoltaic enterprises is of great significance to the improvement of farmers’ income, rural ecology, and productivity.

## 5 Conclusion

In the present study, we tested the usage of the novel EAS (Even-lighting Agrivoltaic System) by two experiments. We measured and investigated the light environments, crop growth, crop yield, and crop quality under various treatments, and calculated the LER and comprehensive economic benefits. The following conclusions were obtained:

- CAS cannot provide a suitable light environment for plants and severely affect the crop yield, i.e., the growth of lettuces under the CAS lags behind those under a natural state for more than 7 days, and the yield is reduced by more than 50%;
- EAS provides uniform illumination for crops, and it increases the irradiation that crops collected in one day by 47.38% compared with the CAS.
- Plants grown under EAS have similar crop yield and quality as those grown in a natural state, the yield reduction of shade-intolerant crops under EAS is within an acceptable range;
- When EAS is combined with supplementary LED lamps, the soluble sugar content of lettuce increased by 72.14% and the nitrate content decreased by 21.51%;
- The EAS has a high LER (averagely 1.64) and thus creates more economic benefits for farmers, allowing their income to be increased averagely by 5.14 times depending on which crops they planted and their corresponding growth time;
- EAS can be the basis of future smart agriculture, many sensors can be installed on it for intelligent control or optimization of agricultural production.

## Supporting information

S1 Table The light spectrum of the supplementary LED lamps (raw data). (XLSX)

S2 Table Variation of the typical light environment (PPFD) of different treatments over time (raw data). (XLSX)

S3 Table Effect of different light conditions on the maximal width of lettuces and the number of lettuce leaves (raw data). (XLSX)

S4 Table The average fresh weight, dry weight, and water content of plants under different light conditions (raw data). (XLSX)

S5 Table Yield per single plant of two cultivation methods in the large-scale field planting experiment (raw data). (XLSX)

S6 Table The raw data of the crop quality of lettuce (soluble protein, soluble sugar, vitamin C, and nitrate) in different treatments. (XLSX)
